# New *Inonotus* Polysaccharides: Characterization and Anticomplementary Activity of *Inonotus rheades* Mycelium Polymers

**DOI:** 10.3390/polym15051257

**Published:** 2023-03-01

**Authors:** Daniil N. Olennikov, Tatyana G. Gornostai

**Affiliations:** 1 Laboratory of Biomedical Research, Institute of General and Experimental Biology, Siberian Division, Russian Academy of Science, Sakh’yanovoy Street 6, 670047 Ulan-Ude, Russia; 2 Laboratory of Plant Physiological Genetics, Siberian Institute of Plant Physiology and Biochemistry, Siberian Division, Russian Academy of Science, Lermontova Street 132, 664033 Irkutsk, Russia

**Keywords:** *Inonotus rheades*, Hymenochaetaceae, mycelium, polysaccharides, galactan, anticomplementary activity

## Abstract

*Inonotus* is a small genus of xylotrophic basidiomycetes and a source of bioactive fungochemicals among which a special place is occupied by polymeric compounds. In this study, polysaccharides that are widespread in Europe, Asia, and North America and a poorly understood fungal species, *I. rheades* (Pers.) Karst. (fox polypore), were investigated. Water-soluble polysaccharides of *I. rheades* mycelium were extracted, purified, and studied using chemical reactions, elemental and monosaccharide analysis, UV–Vis and FTIR spectroscopy, gel permeation chromatography, and linkage analysis. Five homogenic polymers (IRP-1–IRP-5) with molecular weights of 110–1520 kDa were heteropolysaccharides that consist mainly of galactose, glucose, and mannose. The dominant component, IRP-4, was preliminary concluded to be a branched (1→3,6)-linked galactan. Polysaccharides of *I. rheades* inhibited the hemolysis of sensitized sheep erythrocytes by complement from human serum, signifying anticomplementary activity with the greatest effects for the IRP-4 polymer. These findings suggest that *I. rheades* mycelium is a new source of fungal polysaccharides with potential immunomodulatory and anti-inflammatory properties.

## 1. Introduction

Xylotrophic basidiomycetes are a large ecological group of saprotrophic fungi that are often found on wood [1]. The ability to accumulate lignocelluloses is a special feature of xylotrophic basidiomycetes, which include fungi of the genus *Inonotus* consisting of approximately 20 species included in the Basidiomycota division of the Aphyllophorales order and the Hymenochaetaceae family [2]. Currently, basidiomycetes are being investigated as a source of various biologically active compounds. The most promising and widely significant in fungotherapy is the natural sclerotia of *Inonotus obliquus* (Ach. ex Pers.) Pilat or Chaga, which is formed mainly on living birch trunks [3]. Various biological activities of the extracts and compounds from *I. obliquus* have been revealed [4]. Based on Chaga, a number of mushroom-derived medicines have been developed [5]. However, the natural resource of *I. obliquus* is limited, and a wide geographical distribution and various natural conditions have resulted in difficulties in standardizing the raw materials. Many species of the genus *Inonotus* have still been poorly investigated.

**Figure 1:**
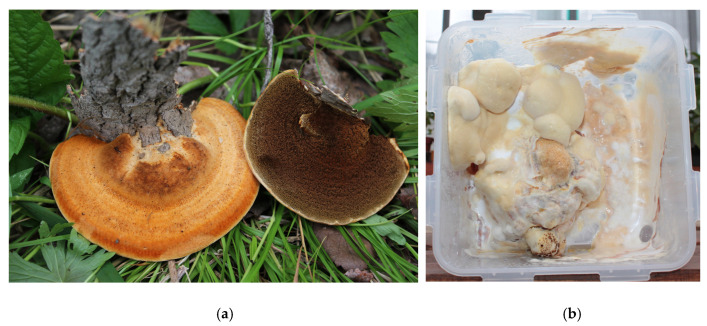
Fruit bodies of *Inonotus rheades* (Pers.) Karst. (fox polypore) in its natural habitat (Kadinskii reserve, Kuitun District, Irkutsk Oblast, Russia) (**a**) and lab-grown *I. rheades* mycelium (**b**).

Among the poorly studied species is *I. rheades* (Pers.) Karst. (fox polypore), a circumboreal species distributed in Europe from north to south, as well as in Asia and North America, and growing more often on species of *Populus tremula* (in Europe) and rarely on *Quercus*, *Salix*, and *Sorbus* (Figure 1) [6]. This annual species has a pileus, is solitary or grows in groups of several pilei, is medium in size, is flat or triangular, and has a thick base, with a uniformly colored upper surface. The old base is rough brown, and the young outer zones are a hairy yellow-brown. The upper surface is dark, and the actively growing edge is pale, soft, and matte. The pores are 2–3 × 1 mm in size and angular or tortuous in shape, with a brown surface. The hyphal system monomitic hyphae are without clamps and are brown without setae. Basidiospores are egg-shaped, in the form of an ellipsoid of 5–6 × 3.5–4 µm [6]. As a rule, this fungal species is considered inedible, although it is not poisonous.

The diverse range of chemical components found in *I. rheades* includes styrylpyrones and bis(styrylpyrones), in particular 1,1-distyrylpyrylethane, *trans*- and *cis*-hispidin, *trans*- and *cis*-bisnoryangonin, phellinins A1 and A2, hypholomine A, 3-bisnoryangonyl-14′-hispidin, hypholomine B, 3,14′-bishispidinyl, rheadinin [7,8], triterpenes as inotodiol, lanosterol, ergosterol peroxide, lupeol, betulin, betulinic acid, betulone, betulonic aldehyde, betulonic acid, and betulinic aldehyde [9], and fatty acids [10] detected in the mycelium. Regarding bioactivity, the extracts of *I. rheades* and pure compounds possess antiglucosidase [9], antioxidant [11], antibacterial, and fungistatic activity [12].

Among the variety of *Inonotus* constituents, polysaccharides are perhaps the most relevant as bioactive compounds of this fungal genus [13]. Structurally, *Inonotus* polysaccharides are homo- and heteropolymers with core chains of α-glucans [14], (1→ 3)- and (1→6)-linked β-glucans [15,16], (1→6)-linked α-galactans, (1 →4)-linked β-xylans [17], and mannans [18]. Various biological studies of *Inonotus* polysaccharides have demonstrated their effectiveness as antitumor [19,20,21], lipid-lowering [22], hypoglycemic [23,24,25], immunomodulatory [26], antioxidant [27], anti-fatigue [28], antiviral [29], gut microbiota-regulated [30], and anti-*Toxoplasma* agents [31]. The polysaccharides of *I. rheades* mycelium have not been previously investigated.

Both the fruiting bodies and mycelium are of practical importance in fungi. The use of pure mycelial mass is relevant for several reasons. First, there is no seasonal dependence on obtaining raw materials compared to fruiting bodies that are collected in nature. It is known that the fruiting period of mushrooms is very limited (1–3 weeks), and some species cannot bear fruit every year [5]. Second, there are no quantitative restrictions for obtaining mycelial mass, which may be associated with the individual characteristics of the fruiting of mushrooms, as well as with changes in climatic and weather conditions. Given the physiological characteristics of fungi and their distribution area, it should be remembered that some fungi do not form a sufficient number of fruiting bodies and may be rare [32]. In nature, the development of a fungal organism also directly depends on climatic factors; for example, during periods of drought, the development of the mycelium slows down, and the formation of primordia and fruiting bodies does not occur [33]. Third, the resulting mycelial mass is microbiologically pure and standardized in quality. Under natural conditions, any organism is subject to contamination, and fungi are no exception. With the multiple actions of various environmental factors, raw materials collected in natural conditions have variations in chemical composition [34]. Fourth, it is more convenient to regulate properties of a mycelium. Using ecological parameters for growing a mycelium, it is possible to regulate its chemical composition; for example, lighting parameters have a direct impact on the morphology of development, physiology, and metabolism of fungi.

As part of the ongoing study of bioactive polysaccharides from Asian fungi [16,35,36,37], this paper presents the results of the isolation and anticomplementary potential of soluble polysaccharides from *I. rheades* mycelium (IRP). The fraction IRP was studied using chemical analysis, elemental analysis, HPLC-UV, ultraviolet–visible and Fourier transform infrared spectroscopy, gel permeation chromatography, and linkage analysis, followed by the bioactivity in vitro assay to determine the ability of biopolymers to interact with the complement cascade reaction.

## 2. Materials and Methods

### 2.1. Fungal Material

The mycelium of the basidiomycete *I. rheades* (Pers.) Bondartsev & Singer, strain 0186, was purchased from the higher basidiomycete culture collection of the Komarov’s Botanical Institute (Russian Academy of Sciences, St. Petersburg, Russia). The culture was stored in the Bioresource Center (Siberian Institute of Plant Physiology and Biochemistry, Irkutsk, Russia). The pure culture was stored on a lean yeast medium at a temperature of 4 °C [38]. The mycelium was grown on wood discs of *Betula pendula* Roth. (Betulaceae) in a sterile plastic 5-L container for 30 days, in KBW 720 stationary chambers (Binder, Tuttlingen, Germany) at 25 ± 1 °C. Light-emitting diodes SMD-5050 (Rubicon, Moscow, Russia) were used as a source of radiation (465–470 nm, luminous flux 12.88 W/m^2^, flux intensity 48.3 µmol/m^2^·s). The resulting pure mycelium mass was dried to an air-dry state at a temperature no higher than 45 °C.

### 2.2. Isolation of Total Polysaccharide Fraction of I. rheades Mycelium (IRP)

The previous method of isolation natural polysaccharides [35,39] was used with modifications. Air-dried mycelium (480 g) was milled, then was extracted with hexane (70 °C; mycelium:hexane ratio 1:10; 2 h; two times), 70% ethanol (80 °C; mycelium:ethanol ratio 1:20; 2 h; five times), and hot water (90 °C; mycelium:water ratio 1:15; 2 h; three times). The water extract was centrifuged (6000 rpm, 30 min), concentrated in vacuo (30 times), and concentrated extract was precipitated using acetone (1:5). After 24 h, the precipitate was separated by centrifugation (9000 rpm, 15 min), suspended in warm distilled water (50 °C, 2 L), and the solution was passed through a polyamide column (0.5 kg; Sigma-Aldrich, cat. No. 02395, St. Louis, MI, USA) followed by cation-exchanging column filtration (KU-2-8, H^+^-form; Eco-Vita, St. Petersburg, Russia; 2 kg). Both columns were eluted with distilled water (3 L). The final water eluate was concentrated to 200 mL (30 °C; in vacuo), and the residue was deproteinated using the Sevag method [35] and protease-assisted deproteination with *Streptomyces griseus* pronase (type XIV, ≥3.5 units/mg; 1 unit per 1 mL of polysaccharide solution; Sigma-Aldrich, cat. No. P5147) [36]. After dialysis in benzoylated dialysis tubes (cut-off of 2 kDa; Sigma-Aldrich, cat. No. D2272) against distilled water (48 h), the non-dialyzed residue was freeze-dried to give a total polysaccharide fraction of *I. rheades* mycelium (IRP) as an off-white powder with a yield of 6.14 g.

### 2.3. Chemical Analysis and Elemental Composition of IRP

Commercial kits were used for spectrophotometric analysis of total carbohydrate content (High Sensitivity Carbohydrate Assay Kit, BioVision, Inc., Milpitas, CA, USA; cat. No. K2049-100), uronic acids (D-Glucuronic/D-Galacturonic Acid Assay Kit, Megazyme, Bray, Ireland; cat. No. K-URONIC), protein (Pierce™ BCA Protein Assay Kit, Thermo Fisher Scientific, Waltham, MA, USA), and phenolics (Phenolic Compounds Assay Kit, Sigma-Aldrich; cat. MAK365). Ash content was determined by the AOAC Official Method^SM^ 942.05 using muffle furnace ignition at 600 °C [40]. Reaction with Yariv reagent was performed by the Lamport et al. assay [41]. All chemical analyses were performed five times, and the data were expressed as the mean value ± standard deviation (S.D.). For the analysis of carbon, hydrogen, oxygen, and nitrogen contents, a 2400 Series II elemental analyzer (Perkin Elmer, Waltham, MA, USA) was used.

### 2.4. Monosaccharide Composition

The monosaccharide composition was determined after hydrolysis with 2 M trifluoroacetic acid [42], 1-phenyl-3-methyl-5-pyrazolone (PMP) labeling, and HPLC-UV separation of PMP-labeled monosaccharides as described previously [39]. Water solutions of reference monosaccharides (mannose, ribose, rhamnose, glucose, galactose, xylose, arabinose, fucose, galacturonic acid, glucuronic acid; all 1 mg/mL) after PMP-labeling were used to build calibration curves (peak area vs. the concentration levels). All analyses were performed in triplicate.

### 2.5. Ultraviolet (UV) and Fourier Transform Infrared (FTIR) Spectroscopy

A Spectrophotometer SF-2000 UV–Vis (OKB Spectr, St. Petersburg, Russia) was used to study the UV spectra of water polysaccharide solutions (5 mg/mL) in the spectral range of 190–1000 nm using a quartz cell (10 mm). To study FTIR spectra, an FT-801 Fourier transform infrared spectrometer (Simex, Novosibirsk, Russia) was used (frequency 4000–600 cm^−1^, 200 scans, 2-cm^−1^ resolution) and samples were tableted with potassium bromide (sample:KBr 1:100).

### 2.6. Molecular Weight Determination and Linkage Analysis

To determine molecular weights, a gel permeation–high performance liquid chromatography procedure was used, with an LCMS 8050 liquid chromatograph coupled with a Shim-pack Diol-150 column (250 × 7.9 mm, ∅ 5 μm; Shimadzu) as described previously [39]. A calibration curve was produced via analysis of dextran series (10–410 kDa; Sigma-Aldrich). All analyses were performed in duplicate. The procedure of linkage analysis was described in a previous study [39], and used methyl iodide methylation, 90% formic acid and 2 M TFA hydrolysis, NaBH_4_ reduction, and acetic anhydride acetylation [43], followed by gas chromatography–mass spectrometry analysis with a 5973N gas chromatograph mass spectrometer (Agilent Technologies, Santa-Clara, CA, USA) equipped with a 6890N mass selective detector, a diffusion pump, and an HP-Innowax capillary column (Agilent Technologies; 30 m × 250 μm × 0.50 μm) [37].

### 2.7. Sephacryl 400HR Gel Fractionation of IRP

The water solution of IRP (5 g in 200 mL) was passed through a Sephacryl 400HR gel column (GE Healthcare, Chicago, IL, USA) and eluted with water, detecting the elution progress spectrophotometrically (λ 190, 260 nm). The separate eluates were collected and acetone-precipitated to give fractions of IRP-1 (290 mg), IRP-2 (555 mg), IRP-3 (565 mg), IRP-4 (3.585 g), and IRP-5 (5 mg).

### 2.8. Partial Hydrolysis of IRP-4 by 0.5% TFA

A sample of IRP-7 (1 g) was incubated at 80 °C with 0.5% TFA (50 mL, 2 h), followed by the TFA elimination in vacuo, dialysis of the residue in dialysis tubes with cut-off 2 kDa (48 h), and freeze-drying of the non-dialyzed sample. The yield of partially hydrolyzed polymer IRP-4d was 320 mg.

### 2.9. Anticomplementary Activity

The anticomplementary activity of polysaccharides was studied using the method of Samuelson et al. [44] with sheep erythrocytes sensitized with rabbit anti-sheep erythrocyte antibodies (BioTrend, Köln, Deutschland, cat. No 113-4139) and human serum with intact complement proteins from healthy adults. Briefly, sheep erythrocytes pre-washed with 0.9% NaCl and veronal buffer (pH 7.2) were sensitized using rabbit anti-sheep erythrocyte antibodies, incubated (37 °C, 30 min), washed, and used to prepare 1% suspension in veronal buffer. Healthy adult human serum with removed antibodies against sheep erythrocytes after absorption on human red blood cells (5 mM EDTA, 0 °C) was diluted with veronal buffer to give serum showing 50% hemolysis. Polysaccharide solutions in in veronal buffer were mixed with serum (1:1), incubated at 37 °C (30 min), mixed with sensitized sheep erythrocytes (2:1), incubated again (30 min), and centrifuged (2000 rpm, 10 min). Absorbance of the sample supernatant was measured at 405 nm. Distilled water was used as negative control, giving 100% lysis, and MPP-2 polysaccharide from *Mentha* × *piperita* was a positive control. Anticomplementary activity was calculated as in the original research [44].

### 2.10. Statistical and Multivariate Analysis

Statistical analyses were performed using one-way analysis of variance, and the significance of the mean difference was determined using Duncan’s multiple range test. Differences at *p* < 0.05 were considered statistically significant. The results are presented as the mean ± S.D.

## 3. Results and Discussion

Water-soluble polysaccharides of *I. obliquus* have been extensively studied and concluded to be bioactive polymers [I7]; therefore, this was the main reason why water was chosen as the extraction medium for isolation of *I. rheades* mycelium polysaccharides. The total polysaccharide fraction (IRP) was isolated from *I. rheades* mycelium with a 1.28% yield, and showed a high carbohydrate content (82.67%) with low levels of protein, uronic acids, phenolics, and ash (Table 1). Negative reactions with iodine, resorcinol, Fehling’s reagent, and Yariv’s reagent indicated a lack of starch [45], fructans [46], galacto/gluco-mannans [47], and arabinogalactan–protein complexes [40], respectively. The elemental composition of IRT showed that the levels for carbon (40.20%), hydrogen (6.68%), and oxygen (53.01%) are typical for neutral polysaccharides [39].

The dominant monosaccharides of IRP were galactose, glucose, and mannose in a ratio of 4.4:1.9:1, and minor components were arabinose, fucose, rhamnose, xylose, and uronic acids with content variations of 0.6–6.5 mol%. The known data on *Inonotus* polysaccharides demonstrated the domination of glucose (68–97 mol%), mannose (2–19 mol%), and galactose (3–6 mol%) in exopolysaccharides of a submerged culture of the *I. obliquus* BELYU1102 strain [48]. Cultivated mycelium of the same strain of *I. obliquus* gave endopolysaccharides containing mannose (23–74 mol%) and glucose (1–15 mol%) [49]. Commercially available wild *I. obliquus* sclerotium was the source of Un-IOPS polysaccharide with levels of glucose, mannose, and galactose at 48, 21, and 17 mol%, respectively [50]. A series of heteropolysaccharide isolated from Chinese *I. obliquus* sclerotium contained glucose (31–40 mol%) and galactose (8–14 mol%) [51]. A submerged culture of *I. levis* gave a polysaccharide fraction that mainly included mannose, galactose, and glucose in a ratio of 3.3:2.3:1 [52]. Thus, given the abovementioned information, galactose, glucose, and mannose are the usual constituents of water-soluble polysaccharides found in the *Inonotus* genus.

**Figure 2:**
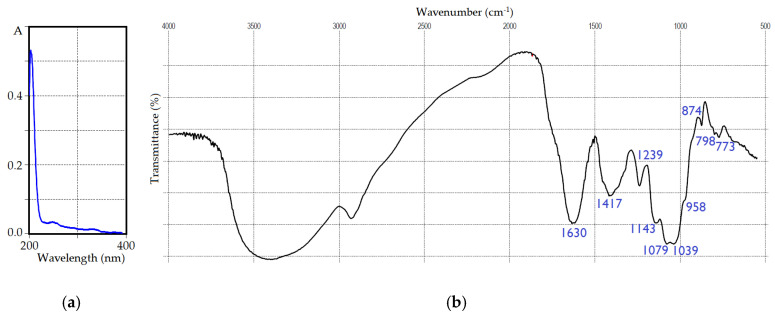
UV (**a**) and FTIR spectra (**b**) of IRP polysaccharide.

The spectral pattern of IRP in the ultra-violet region (Figure 2a) showed no distinct peaks, owing to its low phenolic and protein contents [53,54,55]. FTIR spectral data (Figure 2b) were typical for natural polysaccharides [56] with bands in a skeletal region (<800 cm^−1^) attributed to the vibrations of “breathing” rings at 773 cm^−1^ and sugar cycles at 798 cm^−1^ [57]. The fingerprint region (1200–800 cm^−1^) demonstrated vibrations of β-anomeric galactose/mannose aldopyranose rings at 874 cm^−1^, in-plane bending vibrations of C-OH and C-H linkages of C-1 and symmetric stretching vibrations of glycosidic linkages C-O-C at 958 cm^−1^, stretching vibrations of glycosidic C-O and C-C at 1039 cm^−1^, and in-plane bending vibrations of C-OH fragments at 1079 cm^−1^ [58]. The local symmetry region (1500–1200 cm^−1^) included asymmetric vibrations of C-O-C linkages at 1143 cm^−1^, and scissoring and in-plane bending vibrations of CH and CH_2_ at 1239 and 1417 cm^−1^, respectively [59]. The intense band at 1630 cm^−1^ of scissoring vibrations of hydrated water molecules was found in the double bond stretching region [60]. A broad and strong band of stretching vibrations of hydroxyl groups was observed at 3425 cm^−1^, accompanied by a band of symmetric and asymmetric stretching vibrations of skeletal C-H and CH_2_ fragments [61].

**Figure 3:**
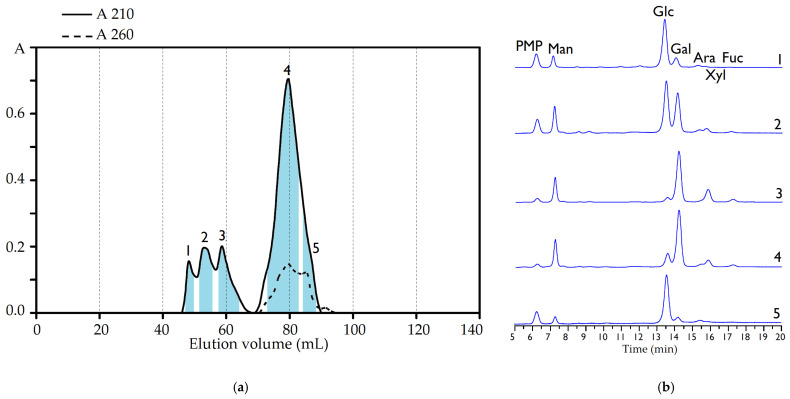
Elution curve of IRP fractions on the Sephacryl 400HR (**a**) and HPLC-UV chromatograms of PMP-labeled monosaccharides in IRP fraction hydrolysates (**b**). IRP fractions: **1**—IRP-1, **2**—IRP-2, **3**—IRP-3, **4**—IRP-4, **5**—IRP-5.

Gel permeation chromatography (Sephacryl 400HR) indicated the heterogeneity of IRP (Figure 3a), and then five homogenic fraction were isolated. Three polymers with low retention were IRP-1, IRP-2, and IRP-3 with molecular weights of 1520, 1150, and 820 kDa, respectively (Table 2). The IRP-1 polymer was characterized by the predominance of glucose, galactose, and mannose in the ratio of 6.1:1.2:1, while IRP-2 also contained glucose, galactose, and mannose as the main monosaccharides but in a different ratio of 2.9:2.2:1. For IRP-3, an increased content of xylose (15.9 mol%) was noted; the ratio of the main monosaccharides galactose, mannose, and xylose was 3.6:1.2:1.

The dominant polymer, IRP-4, with a 71.7% yield of IRP weight, had a molecular weight of 148 kDa and was a heteropolysaccharide containing galactose, mannose, and glucose as the main monosaccharides in a ratio of 4.2:1.4:1. The level of minor monosaccharides accounted for no more than 4.5 mol%. The polymer IRP-5 with the lowest molecular weight (110 kDa) was a glucan with a glucose content of 81.7 mol%. Thus, the complex of water-soluble polysaccharides of *I. rheades* mycelium is a mixture of predominantly glucans (IRP-1, IRP-2, and IRP-5) and galactans (IRP-3 and IRP-4) with varied molecular weights.

Considering the high content of IRP-4 in the IRP total fraction, we studied its structure after methylation and analysis of *O*-methylated alditol acetates using GC–MS. According to methylation data, IRP-4 was a highly branched polysaccharide containing residues of galactose (2,3,4,6-Me_4_-Gal), mannose (2,3,4,6-Me_4_-Man), glucose (2,3,4,6-Me_4_-Glc), xylose (2,3,4-Me_3_-Xyl), arabinose (2,3,4-Me_3_-Ara), and fucose (2,3,4-Me_3_-Fuc) at the non-reducing ends of the chains (Table 3). A high content of 6-*O*-(2,3,4-Me_3_-Gal) and 3,6-di-*O*-substituted galactose residues (2,4-Me_2_-Gal) was revealed, resulting in 56.3% residues in the polymer molecule. The presence of 3-*O*-substituted galactose (2,4,6-Me_3_-Gal) and mannose (3,4,6-Me_3_-Man), as well as 6-*O*-substituted glucose (2,3,4-Me_3_-Glc), was also established. These last residues likely form the side chains of the polysaccharide.

To determine the type of monosaccharides that form the main chain of the polymer molecule, partial hydrolysis was performed, which led to the formation of the IRP-4d fragment with a molecular weight of 25 kDa, the only component of which was galactose.

Analysis of alditol acetates after methylation showed that the main fragment of IRP-4d was 6-*O*-substituted galactose (2,3,4-Me_3_-Gal; 92.6%), which constructs the core of the polymer molecule in the form of a chain [→6)-Gal-(1→]. Thus, the performed studies allowed the preliminary structure of the dominant polymer IRP-4 as (1→6)-linked galactan to be established for the first time, in which more than 60% of galactose residues are substituted at the C-3 position by various carbohydrate fragments.

To study the biological effects of individual polysaccharides of *I. rheades* mycelium, their anticomplementary activities were determined on the model of inhibition of hemolysis of sensitized sheep erythrocytes with complement from human serum [44]. This method allows the study of the ability of polysaccharides to participate in the cascade of reactions of the complement system.

**Figure 4:**
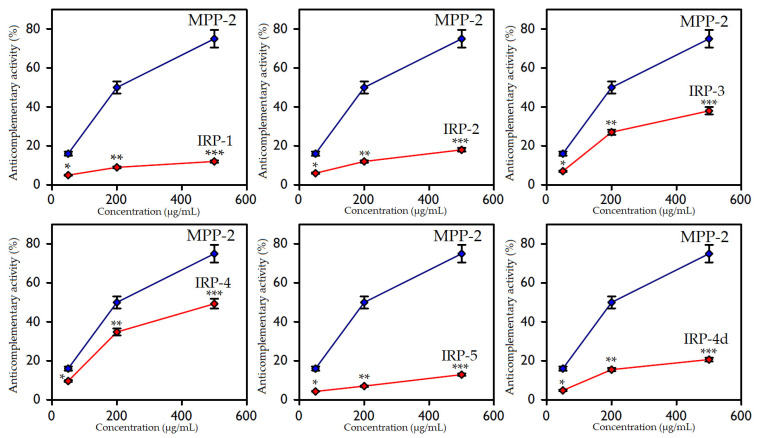
Anticomplementary activity of IRP-1–IRP-5, and IRP-4d polysaccharides. Positive control—MPP-2 polysaccharide from *Mentha* × *piperita*. Asterisks indicate significant difference (*p* < 0.05) vs. the MPP-2 group, 50 μg/mL (*); vs. the MPP-2 group, 200 μg/mL (**); vs. the MPP-2 group, 500 μg/mL (***).

The IRP-1, IRP-2, and IRP-5 polysaccharides had a weak complement-fixing activity, demonstrating binding efficiencies of 12, 18, and 12%, respectively (Figure 4), at concentrations of 500 μg/mL. The binding index for the reference polysaccharide used as a positive control (MPP′-2 polysaccharide from *Mentha* × *piperita*) was 75% [62].

Polysaccharides IRP-4 and IRP-5 showed the highest anti-complementary activities, amounting to 38% and 49% at concentrations of 500 μg/mL, respectively. Thus, considering the chemical composition data of the studied polysaccharides, it can be argued that the biological activities of the components that are glucans (IRP-1, IRP-2, and IRP-5) were significantly lower than those of polymers enriched in galactose, the galactans (IRP-3 and IRP-4). A comparative analysis of the anti-complementary activity of the highly branched galactan IRP-4 and the unbranched fragment of its polysaccharide core IRP-4d indicated the low efficiency of the latter (21% at 500 μg/mL), which showed the important role of the degree of branching for polysaccharide biological activity.

The early knowledge about the structures of *Inonotus* polysaccharides revealed that homogenous polymer IOI-WN of *I. obliquus* sclerotia is (1→3)-linked β-glucan branched at every fifth C-6 position and that the IOE-WN polymer is 3-*O*-methylated (1→6)-linked α-galactan branched at every third galactose fragment [17]. Cultivated mycelium and wild sterile conk of *I. obliquus* gave the mixture of (1→3,6)-linked β-glucans and methylated galactans as minor components [14]. Mixed (1→3,6)-linked galactan consisting of galactose and 3-*O*-methylated galactose in molecular weight was isolated from a submerged culture of *I. levis* together with the minor (1→2,6)-linked mannan [52]. Thus, the isolation of glucans and galactans from *I. rheades* mycelium in our study was not uncommon. Despite the lack of knowledge, the most likely explanation is that glucans and galactans are the principal polysaccharides of the *Inonotus* genus. This makes even more sense if we remember that other fungi of the Hymenochaetaceae family were determined to be sources of polysaccharides; a good example is the *Phellinus* genus enriched with (1→3,6)-linked glucans and (1→3,6)-linked galactans with various fine structures [63].

The complement system is an important part of the body′s immune system, which includes various serum protein components C1–C9 that are activated according to the cascade principle by classical and alternative pathways [64]. The activation of the complement system plays an important role in initiating inflammatory and immune responses including leukocyte activation and degranulation of basophils and mast cells. The anticomplementary potential of natural polysaccharides is known for the polymers isolated from *Echinacea purpurea* (L.) Moench (Asteraceae) [65], *Panax ginseng* C.A. Meyer (Araliaceae) [66], *Abelmoschus esculentus* L. (Malvaceae) [67], and many others [64]. The complement-fixing activity of *Inonotus* polysaccharides has not been previously studied, but it is known that triterpenoids 3β-hydroxy-8,24-dien-21-al and inotodiol and the total melanin fraction of *I. obliquus* sclerotia are potent anticomplementary agents [68].

Anticomplementary potential was reported for (1→3,6)-linked galactans from *Angelica acutiloba* Kitagawa (Apiaceae) [69], *Terminalia macroptera* Guill. & Perr. (Combretaceae) [70], and *Panax* L. plants (Araliaceae) [71]. Arabino-3,6-galactans from *Angelica acutiloba* roots showed potent anticomplementary activity through the classical pathway expressed by the neutral galactan chains to a greater extent than arabinan chains [72]. The elimination of arabinose from arabino-3,6-galactans increased the activity of polysaccharides, demonstrating the essential role of the galactan core for the expression of anticomplementary potential. A similar mode of activity was demonstrated by neutral 3,6-galactans from *Malva verticillata* L. (Malvaceae) seeds [73] and *Angelica gigas* Nakai (Apiaceae) roots [74]. Anticomplementary 3,6-*O*-linked galactan TM4a from *Teucrium viscidum* Blume leaves used the classical and alternative pathways for the activation of the immune system and interacted with C3 and C5 components [75]. Two 4,6-galactans from the herb of *Eclipta prostrata* (L.) L. (Asteraceae) interacted with C1, C2, C4, C5, C7, and C9 components, resulting in the inhibition of the activation of the complement system [76].

However, despite the well-known fact that some natural galactans possessed anticomplementary activity, there are no any studies aimed at the identification of specific targets in the complement system interacting with fungal galactans. Regardless, it may be suggested that 3,6-linked galactan from *I. rheades* mycelium can provide anticomplementary activity through the classical pathway, as preliminary data demonstrated the similarity of its structure to the known bioactive plant polysaccharides. It is most likely that galactan IRP-4 can interact with serum protein components C1–C9, which will be studied later. However, nothing can be concluded regarding the structural similarity of polysaccharides of *I. rheades* mycelium and plant polymers until additional studies of *I. rheades* polysaccharides are completed. The indisputable fact remains that natural galactans are prospective bioactive agents due to considerable evidence. The obtained results showed that galactan IRP-4 is an anticomplementary polysaccharide, indicating its potential immunostimulatory and anti-inflammatory properties.

## 4. Conclusions

Fox polypore or *Inonotus rheades* (Pers.) Karst. is a known xylotrophic basidiomycete occurring in the deciduous forests of Europe and Asia. In this study, we demonstrated that the mycelium of *I. rheades* is able to accumulate a heterogenic complex of five polysaccharides with high amounts of galactose, glucose, and mannose and varied molecular weights for the first time. The dominant polymer of *I. rheades* mycelium IRP-4 with a molecular weight of 148 kDa was purified, and linkage analysis showed that it is a branched (1→3,6)-linked galactan. The results of the bioactivity study showed that *I. rheades* polymers are anticomplementary polysaccharides, and galactan IRP-4 was the most active. Our work demonstrated for the complement-fixing potential of polysaccharides from *Inonotus* fungi the first time. These findings suggest that polysaccharides may be active components of the *I. rheades* mycelium, which, in turn, can be a source of new anticomplementary agents.

## Figures and Tables

**Table 1:** Yield and chemical composition of total polysaccharide fraction of *I. rheades* mycelium (IRP).

Parameter	Value
IRP yield, % of dry mycelium weight	1.28
Carbohydrates, % of IRP weight	82.67 ± 2.14
Protein, % of IRP weight	0.54 ± 0.00
Uronic acids, % of IRP weight	2.40 ± 0.06
Phenolics, % of IRP weight	0.05 ± 0.00
Ash, % of IRP weight	1.24 ± 0.02
Reaction with iodine	negative
Reaction with resorcinol	negative
Reaction with Fehling’s reagent	negative
Reaction with Yariv’s reagent	negative
Elemental composition, %	
Carbon	40.20
Hydrogen	6.68
Oxygen	53.01
Nitrogen	0.11
Monosaccharide composition, mol%	
Ara	3.7
Fuc	6.5
Gal	49.1
Glc	21.5
Man	11.1
Rha	1.2
Xyl	4.7
GalA	1.5
GlcA	0.6

**Table 2:** Yield, molecular weight, and monosaccharide composition of IRP fractions after Sephacryl 400HR separation.

Parameter	IRP-1	IRP-2	IRP-3	IRP-4	IRP-5
Yield, % of IRP weight	5.8	11.1	11.3	71.7	0.1
*M_w_*, kDa	1520 (±1.2%)	1150 (±1.7%)	820 (±1.0%)	148 (±2.9%)	110 (±2.7%)
*M_w_/M_n_*	1.26 (±1.5%)	1.40 (±1.8%)	1.1 (±2.1%)	1.62 (±1.9%)	1.57 (±1.6%)
Monosaccharide composition, mol%					
Ara	2.8	2.2	-	1.7	2.4
Fuc	-	0.8	3.4	1.0	-
Gal	13.8	33.7	57.5	60.9	8.7
Glc	71.0	44.5	4.9	14.5	81.7
Man	11.6	15.3	18.3	20.3	7.1
Rha	0.7	-	-	-	-
Xyl	-	3.4	15.9	1.5	-

**Table 3:** Content of *O*-methyl-alditol acetates in hydrolysates of IRP-4 and IRP-4d polymers.

*O*-Methyl-Alditol Acetate	Content in Hydrolisates, %		Linkage Type
	IRP-4	IRP-4d	
2,3,4,6-Me_4_-Gal	0.9	3.6	Gal-(1→
2,4,6-Me_3_-Gal	7.2		→3)-Gal-(1→
2,3,4-Me_3_-Gal	21.6	92.9	→6)-Gal-(1→
2,4-Me_2_-Gal	34.7	3.5	→3,6)-Gal-(1→
2,3,4,6-Me_4_-Man	17.8		Man-(1→
3,4,6-Me_3_-Man	2.5		→2)-Man-(1→
2,3,4,6-Me_4_-Glc	14.0		Glc-(1→
2,3,4-Me_3_-Glc	0.5		→6)-Glc-(1→
2,3,4-Me_3_-Xyl	0.5		Xyl-(1→
2,3,4-Me_3_-Ara	0.2		Ara-(1→
2,3,4-Me_3_-Fuc	0.1		Fuc-(1→

## Data Availability

Not applicable.
